# Superior dislocation hip with anterior column acetabular fracture - open reduction and internal fixation using a twin incision technique

**Published:** 2012-06-21

**Authors:** Nipun Jindal, Sohan S Sankhala

**Affiliations:** 1 Department of Orthopaedics, Government Medical College and Hospital, Chandigarh, India; 2 Department of Orthopaedics, SMS Medical College and attached hospitals, Jaipur, India

**Keywords:** Anterior column acetabulum, fracture, superior dislocation, hip, ilioinguinal

## Abstract

Superior variety of anterior dislocation of the hip is a rare injury. Its occurrence with acetabular fractures has been documented infrequently. We report a case of superior dislocation of the hip with anterior column acetabular fracture. Open reduction of the hip and internal fixation of the fracture was carried out using a twin incision technique. The course to recovery has been uneventful.

## Background

Anterior dislocation of hip joint is a rare injury accounting for 11-15% of all hip dislocations [1]. Out of the anterior dislocations superior variety is the rarest [2]. Very few reports have described the occurrence of an acetabular fracture along with superior dislocation of the hip. We report a case of anterior column acetabular fracture associated with a superior dislocation of the hip joint. We also report a twin incision technique based on pedicle of circumflex iliac artery to avoid the morbidity of extended approaches for this fracture combination.

## Patient and case presentation

**Figure 1:**
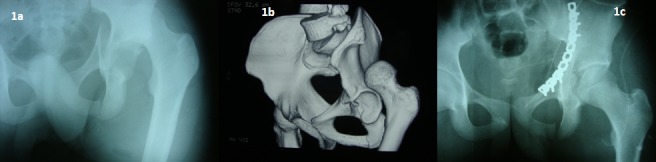
a) Pre operative AP radiograph; Preoperative radiograph clearly showing the anterior dislocation of the hip, disruption of anterior column of the acetabulum and the ischial ramus fracture; b) CT image, 3D CT image depicting the position of the femoral head. Note that there is no bony obstacle to reduction; c) Post operative radiograph, Post operative anteroposterior radiograph showing concentric hip reduction and accurate reduction and fixation of the fracture with a 10 hole 3.5 mm reconstruction plate

A 17 years old male sustained injury while riding a bicycle. His left lower limb was shortened and externally rotated at the hip joint. There was an abnormal protuberance at the supra-acetabular region. There was no associated skeletal injury and the patient was hemodynamically stable. There was no distal neurovascular deficit. The radiographs showed a superior type of dislocation hip with break in the iliopectineal line and fracture of the ischial ramus (Figure 1 (a)). Closed reduction was attempted under sedation in the emergency room but the hip didn't reduce. CT scan of the hip was obtained which showed anterior column fracture at the lateral end of superior pubic ramus and gave away the exact position of the femoral head. No bony obstruction to reduction could be identified on CT images (Figure 1 (b)). Upper tibial skeletal traction was applied with 15 pounds of weight. Reduction was reattempted under general anaesthesia with lateral pull and internal rotation. The head seemed to be lodged in supra-acetabular region with no anterior and posterior translation possible. A fair range of rotation was though present with crepitus.

**Figure 2:**
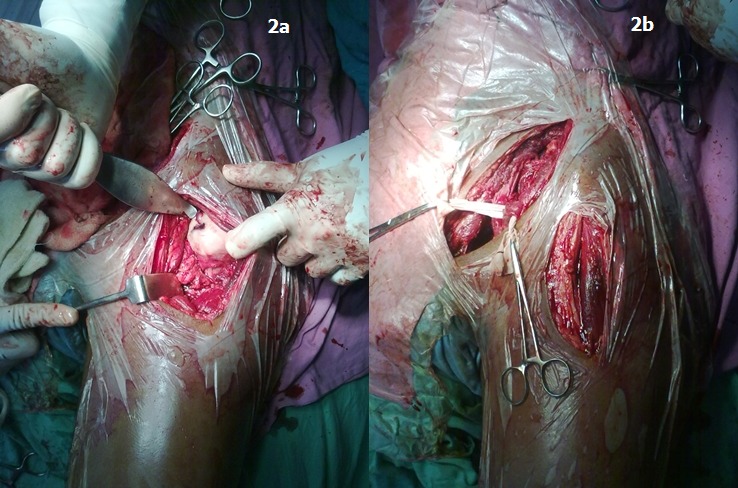
a) Intraoperative image, Intraoperative image showing the completely intact articular surface of the femoral head; b)Twin incision, the twin incision technique as used. Note the intervening skin bridge is the vascular supply zone of the circumflex iliac arteries

Open reduction was then performed for the dislocation. Smith Peterson approach was used for reduction of the hip. It was found that buttonholing of the anterosuperior capsule prevented reduction of hip by closed means. No femoral head fracture was detected nor was any significant chondral damage found (Figure 2 (a)). Hip was reduced and capsule repaired. After the reduction of the hip, anterior column fracture was exposed and fixed with a reconstruction plate using ilioinguinal approach. An intervening skin flap of around six centimetres width was preserved between the two incisions (Figure 2 (b)). The postoperative radiographs showed concentric reduction of the hip with accurate reduction of the fracture (Figure 1 (c)).

The patient was put on mild traction and gradual mobilization and suspension sling exercises were started. He was allowed partial weight bearing in 6 weeks and full weight bearing in 12 weeks. There were no changes of avascular necrosis till the final review. Written informed consent was obtained from the patient for publication of this case report and any accompanying images.

## Discussion

Hip dislocation in adults is the result of very high energy trauma [3]. The classical mechanism of hip dislocation has been described as transmission of forces along the femoral shaft as seen in dashboard injury. The position of the limb at the time of impact dictates the type of dislocation that follows. Anterior dislocation occurs when the limb is abducted and externally rotated. Furthermore flexion at the hip causes inferior type of anterior dislocation while extension causes superior dislocation as described by Epstein et al. The presence of any associated acetabular fractures may be related to the primary impact or due to the dislocating femoral head.

The diagnosis of an isolated superior dislocation is straightforward with lower limb externally rotated and shortened. However associated injuries like femoral neck or shaft fracture may mask the dislocation. These injuries may also be missed in polytraumatised patients who are unconscious or have significant neurotrauma.

The reduction of superior hip dislocation proceeds with strong traction in line of limb axis and gradual internal rotation and flexion of the hip. We however found that flexion at the hip was practically not possible and that lateral pulling on the thigh could be helpful in dislodging the femoral head. Adduction is not recommended during this manoeuvre. Some cases of hip dislocation may not be reduced by closed means. The various attributable causes are femoral head trapped between the medial border of the iliofemoral ligament and the pubocapsular ligament [4], trapping of the femoral head by the iliopsoas [5] and buttonhole defects with tightening of the capsule about the base of the head [6]. We encountered the last described cause for the irreducible dislocation.

The occurrence of acetabular fractures with superior dislocation of hip is exceedingly rare. In a series of 55 patients of anterior hip dislocation described by Epstein et al only two cases of acetabular fractures were mentioned [7]. Mirovsky et al [4] described a fracture involving anterior and superior parts of acetabulum and the anterior inferior iliac spine along with anterior dislocation of hip. Chadha et al [8] reported a case of superior hip dislocation along with posterior acetabular wall fracture. In our case we encountered anterior column fracture. These sporadic reports of acetabular fractures along with superior hip dislocation signify infrequency of these injuries. Also the varied fractures described suggest that the mechanics of the force causing these fractures cannot be generalized.

Operative intervention in such cases normally proceeds with lateral extension of ilioinguinal approach stripping the tensor fascia latae and if required abductor muscles from the lateral aspect of iliac blade. This applies for all the cases where anterior column fixation is required along with dealing with articular pathologies of the hip. In cases of highly comminuted fractures of the anterior column, such an extension is often required for the accurate reduction of fracture fragments. Kloen et al [9] described a T type extension of the ilioinguinal approach for such indications (referred to as modified ilioinguinal approach in the rest of the paper). The advantage exemplified was an achievement of an extensive exposure of the anterior wall and acetabulum without vigorous retraction of the muscles. The lateral femoral cutaneous nerve injury was also reported to be lower but nevertheless occurring in 13% of cases [9].

The twin incision technique employed by us is dissimilar to the modified ilioinguinal approach in three basic ways. Firstly the two incisions in the former are not joined. The intervening soft tissue continues to be supplied by the circumflex iliac vessels [10]. This is of special significance in cases which are predisposed to necrosis of the lateral skin flap formed by the ‘T’ like in critical soft tissue injury, age related skin ischemia or pathological ischemia like in diabetics. Secondly the mechanism of prevention of lateral femoral cutaneous nerve injury is different. In modified ilioinguinal approach, the lateral femoral cutaneous nerve is retracted medially along with the osteotomised anterior superior iliac spine. The nerve may suffer a tractional injury in this type of approach. In the Twin incision technique, the lateral femoral cutaneous nerve virtually goes untouched in the intervening skin pedicle. Thirdly, the twin incision carries out very less muscular elevation off the ilium. This gives a theoretical advantage of decreased incidence of heterotopic ossification as compared to the modified ilioinguinal approach.

The twin incision technique offers less of surgical exposure than the modified ilioinguinal approach but nonetheless is adequate in cases like irreducible anterior dislocation, intraarticular bony fragments and for assisted reduction techniques. For indications like fixation of associated anterior wall fractures, if the technique proves to be inadequate, it can be conveniently converted to the modified ilioinguinal approach.

## Conclusion

Anterior superior hip dislocation is an infrequent but grave injury. The need for early recognition and screening in polytraumatised patient cannot be over emphasized. Prompt closed reduction should be carried out. Although it is usually successful nevertheless, one should not be hesitant in carrying out an open reduction if it is warranted. Associated acetabular fractures and presence of intra articular bony fragments should be carefully looked for. Approaches like the Twin incision technique described here can be used for the surgical management which protect soft tissue, offer sufficient exposure for the fracture and can be converted to extensile approaches without any difficulty.

## References

[1] Epstein HC (1973). Traumatic dislocations of the hip. Clin Orthop..

[2] Epstein HC, Wiss DA (1985). Traumatic anterior dislocation of the hip. Orthopaedics. Orthopedics..

[3] Alonso JE, Volgas DA, Giordano V, Stannard JP (2000). A review of the treatment of hip dislocations associated with acetabular fractures. Clin Orthop..

[4] Mirovsky Y, Fischer S, Hendel D, Halperin N (1988). Traumatic anterior dislocation of the hip joint with fracture of the acetabulum: a case report. J Trauma..

[5] Sherlock DA (1988). Traumatic anterior dislocation of the hip. J Trauma..

[6] Scudese VA (1972). Traumatic anterior hip redislocation – A case report. Clin Orthop..

[7] Epstein HC, Harvey JP (1972). Traumatic anterior dislocations of the hip: management and results, an analysis of fifty five cases. J Bone Joint Surg Am..

[8] Chadha M, Agarwal A, Singh AP (2005). Traumatic anterior dislocation of the hip joint with posterior acetabular wall fracture. Acta Orthop Belg.

[9] Kloen P, Siebenrock KA, Ganz R (2002). Modification of the ilioinguinal approach. J Orthop Trauma.

[10] Penteado CV (1984). Contribution of the superficial and deep circumflex iliac arteries to the blood supply of the anterior third of the iliac crest and adjacent skin. Anat Clin..

